# Colitis-Associated Colorectal Cancer in Patients with Inflammatory Bowel Diseases in a Tertiary Referral Center: A Propensity Score Matching Analysis

**DOI:** 10.3390/jcm11030866

**Published:** 2022-02-07

**Authors:** Kasper Maryńczak, Jakub Włodarczyk, Zofia Sabatowska, Adam Dziki, Łukasz Dziki, Marcin Włodarczyk

**Affiliations:** 1 Department of General and Oncological Surgery, Faculty of Medicine, Medical University of Lodz, 251 Pomorska, 92-213 Lodz, Poland; kasper.marynczak@umed.lodz.pl (K.M.); jakub.wlodarczyk@stud.umed.lodz.pl (J.W.); zofia.sabatowska@umed.lodz.pl (Z.S.); lukasz.dziki@umed.lodz.pl (Ł.D.); 2 Department of Biochemistry, Medical University of Lodz, Mazowiecka 6/8, 92-215 Lodz, Poland; 3 Department of General and Colorectal Surgery, Medical University of Lodz, 113 Żeromskiego, 90-624 Lodz, Poland; adam.dziki@umed.lodz.pl

**Keywords:** colitis-associated colorectal cancer, inflammatory bowel diseases, colorectal cancer

## Abstract

Background: Inflammatory bowel disease (IBD) is a risk factor in developing colitis-associated colorectal cancer (CA-CRC). CA-CRC causes the death of about 15% IBD patients and the risk is 1.5–2.4 fold higher among IBD sufferers than in the general population. The dysplasia CA-CRC develops in a different mechanism in comparison to sporadic colorectal cancer (CRC). This study aimed at evaluating the surgical treatment and its outcomes as well as 5-year survival rates in the CA-CRC and sporadic CRC patients. Materials and methods: This single-center, retrospective, propensity score-matched case-control study was conducted with 2204 patients operated on due to primary CRC, who were hospitalized from 2003 to 2019. The CA-CRC group consisted of 49 patients with CRC in the course of IBD. The sporadic CRC group was selected with the propensity score matching technique and comprised 98 patients with sporadic CRC who did not have clinical or histopathological features characteristic for IBD. Results: CA-CRC is characterized by a more aggressive clinical course. Surgical treatment of CA-CRC involves more palliative operations and is related with a higher risk of perioperative and postoperative complications. Further studies of CA-CRC risk factor stratification and the development of molecular markers hold promise in reducing CRC in IBD patients in the future were warranted.

## 1. Introduction

Currently, many risk factors for the development of colorectal cancer (CRC) have been identified and well established. Inflammatory bowel diseases (IBD) are confirmed as significant factor in development of CRC, especially involving young onset with more aggressive or advanced disease.

Approximately only 1–2% CRCs are related with the course of IBD. The most important and well-recognized risk factors for Colitis-associated cancer colorectal cancer (CA-CRC) are duration and extent of intestinal inflammatory lesions [1,2,3,4]. The genetic factors coupled with the chronic inflammatory process in the colonic mucosa of IBD patients are hypothesized to play a significant role in carcinogenesis, and influencing the inflammatory process could lower this continuous process of inflammation related carcinogenesis in colonic tissue [5,6,7]. In contrast to occasional CRC, CA-CRC occurs at a younger age, it is more often located proximally, and synchronic lesions are also more frequent [6]. CA-CRC is one of the most severe complications of IBD and constitutes the cause of death in 10–15% of IBD patients [8]. CRC development in course of ulcerative colitis is known as a life-threating condition. Therefore, the increased colonoscopy and—in some cases—elective colectomy is advised [9].

The survivability depends heavily on the stage of the disease, according to the TNM classification, developed by the AJCC and, to a lesser extent, on the localization of the tumor in the intestine [10]. The incidence of colitis associated CRC is proven to be reduced by early surgical resection, as well as appropriate screening. The analyses of oncologic results after curative surgeries in patients with CA-CRC compared with matched groups of patients with sporadic CRC is somewhat conflicted [7,11].

Accordingly, this study aimed to evaluate the surgical treatment and its outcomes as well as 5-year survival rates in CA-CRC and a matched group of sporadic CRC patients.

## 2. Materials and Methods

This single-center, retrospective, propensity score-matched case-control study has been conducted on 49 consecutive IBD patients operated on due to CRC, who were hospitalized from 2003 to 2019. The study group consisted of IBD patients who underwent the surgical procedure due to CRC. Only patients with both diagnoses (IBD and CRC) confirmed on final histopathological assessment of resected surgical specimen were included. The control group (n = 98) has been selected from sporadic CRC patients operated on. The propensity score matching (PSM) has been performed to exclude bias resulting from potential confounding factors. Propensity scores were estimated using logistic regression with a matching ratio of 1:2. The following covariates have been included in the regression model: age, sex, BMI index, comorbidities, neoadjuvant treatment, histopathological type of CRC, and the primary location of tumor.

The stage of tumor has been presented using the TNM scale (tumor-node-metastasis, the American Joint Committee on Cancer). According to the current NCCN guidelines, routine pre-operative work-up has been completed for all enrolled CRC patients. This included physical examination, total colonoscopy (unless an obstruction was present), abdominal computed tomography (CT), chest X-ray, complete blood count, carcinoembryonic antigen (CEA), and carbohydrate antigen 19-9 (Ca19-9).

The data for the study was collected using a retrospective analysis of medical documentation, surgical protocols, histopathological findings, and information from the hospital outpatient clinic. Locoregional recurrence has been defined as recurrent disease within the original tumor location (perianastomotic, peritoneum, retroperitoneum, and pericolic mesenteric lymph nodes), while distant recurrence included all recurrent diseases at non-regional sites, such as the liver or lungs. Data on long-term outcomes has been collected by reviewing patient’s records from the hospital outpatient clinic, where the follow-ups were continued. The data has been analyzed for age, sex, BMI, clinical symptoms, type of conducted diagnostics, histopathological findings, type of implemented treatment, intra- and post-operative complications, and finally early- and long-term treatment results.

### 2.1. Ethical Considerations

The study has been conducted in accordance with the ethical principles of the 1975 Declaration of Helsinki and the study protocol was approved by the Committee of Bioethics of Medical University of Lodz, Poland (RNN/463/12/KB).

### 2.2. Statistical Analysis

The data gathered in the study has been analyzed with the statistical package Statistica 13.1 (StatSoft, Inc., Tulsa, OK, USA). The analyzed results have been presented as a mean standard deviation regarding continuous variables and as numbers and percentage referring to categorical variables. The estimation of normality of distribution of the examined quantitative parameters has been executed with the W Shapiro–Wilk test. The comparisons of the study groups have been performed with the Student’s *t*-test (or nonparametric the Mann–Whitney test, depending on the distribution of variables) and the chi-squared test (or Fischer test). While comparing more than two variables in the normal distribution and equal variances the ANOVA variance analysis has been used; otherwise, or in the case of categorical variables the Kruskal–Wallis test has been used. The survival analysis has been executed using the Kaplan–Meier statistics, and the statistical significance of the differences between the two groups has been evaluated with the log-rank test. In all the analyses the probability value *p* < 0.05 has been considered statistically significant.

## 3. Results

A total sample of 147 patients who underwent colorectal resection due to CRC from January 2003 and December 2019 at the Department of General and Colorectal Surgery were enrolled in our study: 65 men (44.2%) and 82 women (55.8%). Study group involved 49 patients, 15 of them were diagnosed with Crohn’s disease, and 34 with ulcerative colitis. The sporadic cancer group consisted of 98 patients. The baseline characteristics and sociodemographic data of all subjects enrolled in the study are presented in Table 1.

**Figure 1:**
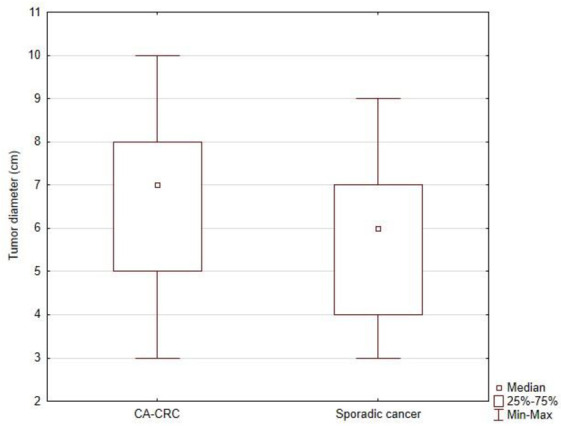
Comparison of tumor diameter (cm) between study group (CA-CRC) and sporadic CRC group.

**Figure 2:**
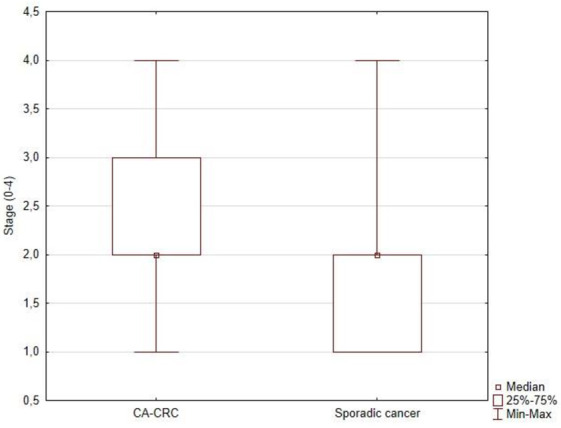
Stage of CRC according to the TNM scale (tumor-node-metastasis, the American Joint Committee on Cancer) in CA-CRC and sporadic cancer groups.

Diameter of tumor in IBD-related CRC patients were significantly higher than in sporadic cancer group (6.82 ± 2.04 cm vs. 5.87 ± 1.76 cm; *p* = 0.006) (Figure 1). Analysis of the histopathological examination of resected tumors revealed that patients in study group had significantly more advance cancer stage than in the sporadic cancer group (mean values: 2.37 ± 0.86 vs. 1.98 ± 0.79; *p* = 0.008) (Figure 2).

Analysis also included the location of the primary tumor. The rectum was the most common primary tumor location in both groups (32.7% in study group; 32.6% in control group). The second most common primary tumor was the descending colon. Specific presentation of tumor localization can be found in Table 2.

In the group of patients with CA-CRC, the tumor infiltrates the surrounding anatomical structures more frequently (20.4%) compared to the control group (7.1%; *p* = 0.027). In both studied groups of patients, the infiltration was most often related to the small intestine. In the study group, the infiltration of the small intestine was 10.2% and in the control group it was 3.1%. The organs affected by neoplastic process in detail are presented in Table 3.

Analysis of surgical treatment outcomes revealed that, statistically, more often tumors in the study group were characterized as unresectable (28.6% vs. 14.3%; *p* = 0.037). However, no significant statistical differences were observed in the percentage of R0, R1, and R2 resections in the analyzed groups (*p* = 0.125). R0 resections were performed in 91.4% of patients in sporadic CRC group and 97.6% in the study group. R1 resections were performed in 8.6% of patients in the control group and 2.4% in the study group.

The early postoperative complications in both groups of patients were also assessed. Early postoperative complications were significantly more frequent in patients with CRC in the course of IBD (38.8% vs. 22.4%; *p* = 0.037). In patients with sporadic CRC, the most frequently observed complication was postoperative bleeding (8.2%) and postoperative wound evacuation (6.1%). On the other hand, in the case of patients with CRC with IBD, 16.3% of patients had an evacuation of a postoperative wound. There was also a relatively frequent leakage of the intestinal anastomosis, which affected 10.2% of patients whose cancer developed due to an inflammatory disease. The third most frequent complication in this group was postoperative bleeding (6.1%). Surgical site infection was the least frequently observed complication in the control and study groups. In both groups, there was also 1 case of perioperative death. Nevertheless, the analysis of differences in the case of specific types of postoperative complications did not reveal statistically significant differences between the control group and the study group.

**Figure 3:**
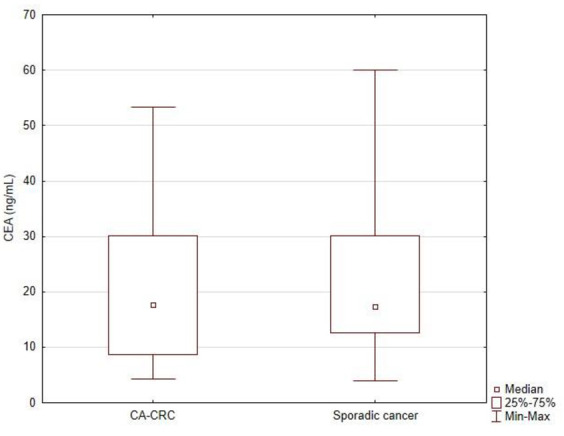
Comparison of CEA (ng/mL) level between study group (CA-CRC) and sporadic CRC group.

**Figure 4:**
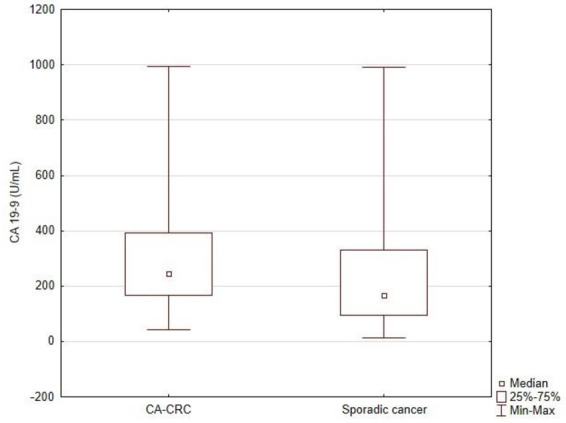
Comparison of CA 19-9 (U/mL) level between study group (CA-CRC) and sporadic CRC group.

The level of tumor markers CEA and Ca 19-9 in the serum of patients from both groups was analyzed. The study showed no statistically significant differences in serum CEA levels between the group of patients with CA-CRC as compared to the group of patients with sporadic cancer (21.1 ± 14.9 ng/mL vs. 22.6 ± 14.6 ng/mL; *p* = 0.363; Figure 3). However, the study showed a statistically significantly higher serum Ca19-9 level in the study group of patients (327.0 ± 237.9 U/mL vs. 254.9 ± 230.9 U/mL; *p* = 0.007; Figure 4).

**Figure 5:**
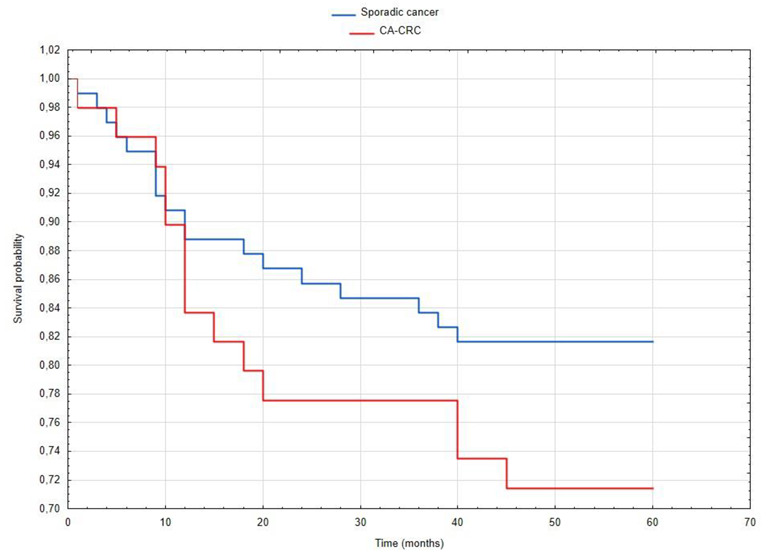
Kaplan–Meier survival curve of CA-CRC group and sporadic CRC group.

The analysis of survival after surgery in the group of patients with CA-CRC and in the group with sporadic CRC showed that the overall 5-year survival for the first group was 71% and for the second group—82%. Comparing the groups, statistically significant differences were obtained—in the analysis with the log rank test the result was Chi2 = 11.8081 (*p* = 0.00273). Plotted survival curves according to the Kaplan–Meier survival function estimation are shown in Figure 5.

## 4. Discussion

The increased risk of CRC in IBD is well documented but whether or not there are differences between sporadic CRC and IBD is not fully understood. The purpose of this study was to compare the course of those two types of CRC and the results of their surgical treatment.

Out of all patients (2204) operated in 2003–2019, only 49 (2%) patients were qualified to the study group—the group of patients with CRC in the course of IBD (CAC). Studies by Gade et al., indicate a similar percentage of patients with CRC in course of inflammatory bowel diseases [12].

Due to implemented propensity score-match analysis average age of patients with both sporadic CRC and patients with CAC was 41 years, which is relatively low. It should be emphasized that such young age among patients with CRC is alarming. Moreover, numerous publications indicate an upward trend in CRC cases among increasingly younger patients [13,14,15]. Regarding the age comparison between groups of patients with CAC and sporadic CRC indicate that CAC affects patients at a younger age than from the second group [12,16,17,18,19]). The low mean age of patients with CRC reported in this study is particularly important considering screening tests. Early detection of CRC undoubtedly influences the success of treatment—as many as 90% of patients with early-stage cancer have a chance of 5-year survival [20]. This emphasizes the role of early diagnostics, especially colonoscopy and constant consideration of new reports in the applied methodologies, techniques, and recommendations, play an important role here. Additionally, screening should be considered even earlier than current recommended age. Moreover, many studies confirm that colonoscopic surveillance in IBD contributes to more early-stage detection and a reduction in colorectal cancer-related deaths [21,22,23].

Our study has shown that patients with IBD were statistically more likely to come from cities than patients with sporadic CRC. The obtained results are confirmed by many publications examining the influence of progressive westernization of life on the dynamics of IBD and colon cancer. About 60% of CRC cases occur in developed countries [17,24]. In countries with low socioeconomic status, fewer cases are observed [25]. CAC and IBD have common features regarding the demographical distribution where both diseases have high incidence in developed and western countries. Moreover, the incidence rate increases in countries adopting western lifestyles [26]. It is suggested that the increase in IBD incidence is primarily the result of urbanization. It is speculated that the reasons for this phenomenon lie in the growth of industrialization, high hygienic and sanitary standards, frequent use of antibiotics and preventive vaccinations, as well as highly processed food [27].

CAC was characterized by a greater degree of advancement expressed by a larger tumor diameter (6.82 ± 2.04 cm in the study group, 5.87 ± 1.76 cm in the control group; *p* = 0.006), higher staging (2.37 ± 0.86 in the study group, 1.98 ± 0.79 in the control group; *p* = 0.008), and more frequent invasion of accompanying anatomical structures. In studies comparing sporadic CRC with CAC, the latter is more advanced. Most cancers in IBD are characterized by no or poor differentiation of lesions [28,29]. The diagnosis of CRC in patients with IBD is often at an advanced stage. It may be caused by delayed diagnosis of a developing cancer due to the attribution of symptoms such as bleeding or changes in bowel rhythm to inflammatory disease [30]. Moreover, patients with IBD are more likely to have multifocal lesions compared to patients with sporadic CRC [18,31].

Patients with CAC had significantly higher levels of Ca 19-9 antigen (327.0 ± 237.9 U/mL in the study group, 254.9 ± 230.9 U/mL in the control group; *p* = 0.007). There was no difference in the level of CEA antigen in the study and control groups. In the test group, this level was 21.1 ng/mL, while in the control group it was slightly higher than 22.6 ng/mL. The predictive value of the Ca19-9 antigen in the assessment of recurrences and overall survivability as well as other diseases developing on the basis of IBD definitely deserves attention [32,33,34].

Five-year survivability was lower in patients with IBD in relation to 5-year survivability in patients with sporadic CRC. Lower survivability of patients with CAC was also observed in other studies and most recent meta-analysis [19,28,29,35,36,37]. Klos et al. observed significantly lower overall 3-year survivability for patients with IBD. However, no differences were noted for 1, 5, or 10 years after surgery. The reason for lower survivability of patients with CAC can presumably be indicated in the unfavorable, higher stage of the disease compared with sporadic CRC [38].

The process of carcinogenesis in course of IBD is complex and not fully understood. However, it is certainly known that inflammation plays crucial role both in IBD mechanism and tumorigenesis. Various cytokines have been proposed to take part in carcinogenesis and inflammatory processes. E.g. recently De Simone et al. stated that interleukin-21 promotes a protumorigenic inflammatory circuit in the development of CRC [39]. Whereas interleukin-25, which is proven to be deficiently synthesized in IBD patients, seems to have beneficial effects and inhibits the tissue-damaging immune response in gut inflammation [40]. CAC surgeries were characterized by extended resections of the colon. In ulcerative colitis patients restorative total coloproctectomy with ileal pouch-anal anastomosis (IPAA), offers in the majority of patients a complete removal of the diseased or potentially diseased mucosa, an effective prevention and treatment of colorectal cancer, and an unchanged body image with no stoma and a preserved anal route of defecation. For this reason, IPAA is today the gold standard for UC patients with CAC. In Crohn’s disease current guidelines recommend pan-proctocolectomy in CAC patients [41,42]. Recent studies presented that less extended resections, involving segmental colonic resections, offer similar long-term outcomes to more extensive surgery in patients with Crohn’s disease and ulcerative colitis with endoscopic remission of intestinal lesions.

Regarding the present study, it should be emphasized that acquiring great number of CAC patients is extremely difficult. Presented study was biased due to involvement of both CD and UC patients in analyzed study group. The increasing incidence of IBD it is an issue that requires special attention. Novel diagnostic tools and predictive markers are crucial to enable earlier diagnosis in order to implement quick therapy and decrease the mortality and morbidity of CAC [43]. Moreover, the impact of recent immunosuppressive therapies of IBD on inflammation and carcinogenesis needs to be addressed. Therefore, further longitudinal and multicenter studies are necessary.

## 5. Conclusions

To summarize, the results of the conducted research confirm that CAC differs from the sporadic cancer of the colon. The most important observation is its higher degree of advancement, which in this study was manifested by a larger diameter of the tumor, higher staging, and more frequent infiltration. This affects the course and effectiveness of surgical treatment, aggravating the prognosis of patients, and highlights the role of early diagnosis of CRC, especially in the group of patients with IBD. It is also noteworthy that the observed increased level of Ca19-9 may be helpful in the early diagnosis of CRC in the course of IBD.

## Figures and Tables

**Table 1:** The baseline characteristics and sociodemographic data of all subjects enrolled in the study.

		Sporadic Colorectal Cancer	Colitis-Associated Colorectal Cancer	*p*-Value
Number of patients		98	49	
Age		41.61 ± 5.27	41.32 ± 6.88	0.901
Sex	Women, n (%)	54 (55.1%)	28 (57.1%)	0.814
	Men, n (%)	44 (44.9%)	21 (42.9%)	
Smoking, n (%)		25 (25.5%)	13 (26.5%)	0.894
BMI, kg/m^2^		24.1 ± 3.74	23.2 ± 3.83	0.233
Education	Primary, n (%)	35 (20.4%)	14 (28.6%)	0.368
	Secondary, n (%)	43 (43.9%)	20 (40.8%)	
	University, n (%)	20 (35.7%)	15 (30.6%)	
Domicile	Town, n (%)	54 (55.1%)	36 (73.5%)	0.031
	Village, n (%)	44 (44.9%)	13 (26.5%)	

**Table 2:** Tumor localization.

Tumor Localization	Sporadic Colorectal Cancer n = 98	Colitis-Associated Colorectal Cancer n = 49	*p*-Value
Rectum	32 (32.6%)	16 (32.7%)	1
Sigmoid	13 (13.3%)	8 (16.3%)	0.617
Descending colon	18 (18.4%)	9 (18.4%)	1
Transverse colon	11 (11.2%)	5 (10.2%)	0.851
Ascending colon	10 (10.2%)	5 (10.2%)	1
Cecum	14 (14.3%)	6 (12.2%)	0.734

**Table 3:** Colorectal cancer invasion on other organs.

	Sporadic Colorectal Cancer	Colitis-Associated Colorectal Cancer	*p*-Value
Small intestine	3 (3.1%)	5 (10.2%)	0.072
Bladder	1 (1%)	2 (4.1%)	0.216
Reproductive organs	1 (1%)	1 (2%)	0.615
Abdominal wall	2 (2%)	2 (4.1%)	0.473
Summary, n (%)	7 (7.1%)	10 (20.4%)	0.018

## Data Availability

The data presented in this study are available on request from the corresponding author. The data are not publicly available due to privacy issues.
